# A New Advanced and Validated Method for the Determination of Potentially Toxic Metals and Trace and Ultra-Trace Elements in Peritoneal Fluid to Assess the Health Risks of Women with Gynecological Pathologies

**DOI:** 10.3390/toxics11050399

**Published:** 2023-04-23

**Authors:** Andrea López-Botella, Raquel Sánchez, José-Luis Todolí-Torró, María José Gómez-Torres, Irene Velasco, Maribel Acién

**Affiliations:** 1 Service of Obstetrics and Gynecology, Unit of Human Reproduction, FISABIO—San Juan University Hospital, Carretera Alicante-Valencia s/n, 03550 Alicante, Spain; andrea.lopez@ua.es (A.L.-B.); macien@umh.es (M.A.); 2 Biotechnology Department, Faculty of Sciences, University of Alicante, Carretera San Vicente del Raspeig s/n, 03690 Alicante, Spain; mjose.gomez@ua.es; 3 Department of Analytical Chemistry, Nutrition and Food Sciences, University of Alicante, Carretera San Vicente del Raspeig s/n, 03690 Alicante, Spain; r.sanchez@ua.es; 4 Gynecology Division, Faculty of Medicine, Miguel Hernández University, Carretera Alicante-Valencia s/n, 03550 Alicante, Spain

**Keywords:** ICP-MS/MS, potentially toxic elements, peritoneal fluid, gynecological pathologies, health effects, environmental contamination

## Abstract

Toxic metals found in the environment have been linked to female infertility and gynecological illnesses. Reliable analytical methods, such as inductively coupled plasma tandem mass spectrometry (ICP-MS/MS), are necessary to determine the elemental composition of biological samples. Currently, the multielemental profile of peritoneal fluid (PF) samples has not yet been established. Due to the complexity of the PF matrix, an ICP-MS/MS-based method has been optimized to mitigate matrix effects and spectral interferences. A dilution factor of 1:4 was the best option to mitigate matrix effects while keeping sensitivity at an appropriate level. A collision gas (He) was useful to lower the extent of spectral interferences for ^56^Fe, ^52^Cr, ^63^Cu, and ^68^Zn. An intermediate validation test was performed to evaluate accuracy, achieving recoveries ranging from 90 to 110%. The method was validated in terms of intermediate precision, reproducibility, and trueness, with an expanded uncertainty lower than 15%. Afterward, it was applied to perform the multielemental analysis of 20 PF samples. The concentrations for major analytes were up to 151 µg L^−1^. Meanwhile,^209^Bi, ^111^Cd, ^52^Cr, ^55^Mn, ^95^Mo, ^60^Ni, ^208^Pb, ^118^Sn, and ^51^V were present at concentrations included within the 1–10 µg L^−1^ range, while ^59^Co and ^139^La were found at concentrations below 1 µg L^−1^.

## 1. Introduction

The chemical elements present in cells and tissues are divided into structural elements (or macroelements) and trace elements [1]. According to the WHO, trace elements can be classified into three groups based on their biological role in the body: essential trace elements, which include iodine (I), zinc (Zn), selenium (Se), copper (Cu), molybdenum (Mo), and chromium (Cr); probably essential trace elements, which include manganese (Mn), silicon (Si), nickel (Ni), boron (B), and vanadium (V); and potentially toxic elements, some of which may have essential biochemical functions, such as fluorine (F), lead (Pb), cadmium (Cd), mercury (Hg), arsenic (As), aluminum (Al), lithium (Li), and tin (Sn). 

Today, a group of elements known as “heavy metals” are studied because of the concerns about their potential harmful effects on human health. Heavy metals are defined as naturally occurring elements that have a high atomic weight and a density at least five times greater than that of water [2]. There are about 51 different elements that could be included in this category. However, the imprecision of this term has led to controversy, and in environmental research studies the term “potentially toxic metal elements” (PTMEs) is preferred, as it is more precise [3,4]. These elements are widely distributed in the environment due to multiple industrial, domestic, agricultural, medical, and technological applications [5]. The environment, lifestyle, and occupational exposure are factors that may contribute to human exposure to PTMEs [6]. 

Recent literature has linked environmental contaminants to human reproductive health concerns, affecting the ability to conceive healthy offspring [7]. Although the contribution of environmental exposure to infertility remains unclear, there is plenty of evidence suggesting that it could negatively affect fertility, as studies involving occupational exposure and animal experiments have demonstrated [8]. Some environmental contaminants, such as PTMEs [9], are considered to be endocrine disrupting compounds (EDCs). EDCs are chemical pollutants capable of interacting with the functions of endogenous hormones, thereby disrupting metabolic pathways [10]. For instance, cadmium (Cd) is a PTME that activates the estrogen receptor alpha (Erα), leading to estrogenic activity [6,11]. Thus, it may be related to the development of estrogen-dependent diseases [6]. Cadmium’s estrogenic properties are thought to contribute to the etiology of leiomyoma [12]. However, the available data on Cd exposure and its association with multiple adverse reproductive health outcomes in women remain controversial and require further investigation [12]. In other gynecological diseases, such as endometrial (END-P) and exocervical polyps (EX-P), researchers found a significant accumulation of Al, Cd, Ni, and Pb (END-P) or Al, Cd, and Cu (EX-P) [13]. Women with END-P were found to have higher Cu/Zn serum ratio levels than controls [14], while decreased Zn and increased Pb levels in the blood were associated with endometriosis in Asian women [15]. Illnesses such as breast cancer, endometriosis, endometrial cancer, menstrual disorders, spontaneous abortions, pre-term deliveries, and stillbirths can be related to exposure to PTMEs [16]. Although the number of studies on the topic is limited, the available evidence suggests that PTMEs could increase the risk of female infertility [16,17]. 

Analytical data studying the accumulation of PTMEs in organs or tissues and biological fluids such as whole blood, serum, plasma, peritoneal fluid (PF), and follicular fluid—among others—can provide useful information about their correlation with the quality of the environment, lifestyle, and diet. Thus, sensitive analytical low-volume-consuming procedures must be developed to achieve this goal and to provide an individualized patient study, since there is evidence of the impact of the increasing environmental load of PTMEs on female fertility. PF, in particular, is an extracellular fluid, representing plasma ultrafiltration [18] and ovarian exudation, caused by increased permeability. This fluid suffers cyclic variations in both volume and steroid hormone levels, with the latter being higher in PF than in plasma [19]. Peritoneal fluid (PF) is a normal, sterile, lubricating fluid found in the pelvic cavity to reduce the friction between the organs and the abdominal wall. It is present in a small and variable volume (2–10 mL) in healthy people, both male and female. However, multiple conditions—such as liver dysfunction, infection, cancer, or inflammatory processes—can lead to an abnormal accumulation of this fluid [20]. In women, PF is produced by normally functioning ovaries, with cyclic variation in volume, and is reabsorbed by the mesothelial cells of the peritoneal cavity. However, ovary-related diseases—such as epithelial or metastatic ovarian cancer, benign ovarian fibroma, stromal hyperplasia, primary peritoneal serous carcinoma, endometriosis, or ruptured ovarian functional cysts, among others—should be kept in mind when women are found to have an excess of PF [20]. PF biomarkers could be useful for the diagnosis and prognosis of gynecological pathologies, as well as for the prediction of the medical response to a given treatment, as this fluid contains specific biomarkers of gynecological diseases [21]. 

Inductively coupled plasma optical emission spectrometry (ICP-OES) [22,23] and inductively coupled plasma mass spectrometry (ICP-MS) [24,25] are analytical techniques that are suitable for multielemental analysis of clinical samples. ICP-MS is considered to be the most suitable approach due to its high sensitivity and ability to provide extremely low limits of quantification for numerous analytes. Although its use is well documented on a wide variety of biological samples, the proper use of ICP-MS requires a good knowledge of the different types of spectral and non-spectral interferences, especially when dealing with biological samples [26]. Otherwise, inaccurate results can be obtained. To address this issue, inductively coupled plasma tandem mass spectrometry (ICP-MS/MS) has emerged as a useful method for overcoming spectral interferences caused by the main sample components [27]. 

This article presents a newly validated method for the detection and quantification of PTMEs, trace metals, and ultra-trace metals in peritoneal fluid (PF) samples from female patients with gynecological pathologies. To the best of our knowledge, this is the first time that ICP-MS/MS has been applied to the analysis of these kinds of sample. The method was optimized in terms of minimization of the consumed sample volume, removal of interferences, and shortening of the analysis time.

## 2. Materials and Methods

### 2.1. Instrumentation and Equipment

An Agilent 8900 ICP-MS/MS (Agilent Technologies, Santa Clara, CA, USA) equipped with an octopole reaction system (ORS) and axial acceleration technology was used throughout the present study. A micromist nebulizer (Glass Expansion, Weilburg, Germany) and a thermostatted double-pass spray chamber (Glass Expansion) were used to introduce the PF samples into the plasma. Table S1 in the Supplementary Materials summarizes the instrumental operating conditions.

### 2.2. Study Group and Sample Collection

Twenty PF samples were randomized and double-blinded for selection and collection between June 2020 and September 2022, from female patients recruited from the gynecology division of San Juan de Alicante University Hospital, Spain, during a surgical procedure in the operating room. The age of the female cohort ranged from 13 to 43 years (35.10 ± 8.53), and their body mass index ranged from 18.4 to 29.7 (22.30 ± 3.27) kg m^−2^. Each patient provided their informed consent after receiving a detailed explanation of the study. Ethical approval was granted by the Institutional Review Board of the Hospital Universitario San Juan de Alicante (Committee protocol code: 19/344, date of approval: 17 December 2019).

Samples were obtained by laparoscopy and collected by aspiration into sterile syringes. Then, the samples were filtered through a 20 µm filter to remove fibrin and cell aggregates and transferred to a glass centrifuge tube. After centrifugation (600× *g*, 10 min), the supernatants were stored in the dark at −20 °C until analysis.

### 2.3. Reagents and Solutions

Calibration curves were obtained from six standards prepared by proper dilution of a 100 mg L^−1^ multielemental stock solution (SCP33MS) purchased form SCP SCIENCE (Clark Graha, Baie D’Urfé, Canada). SCP33MS contained the following analytes: arsenic (As), barium (Ba), bismuth (Bi), cadmium (Cd), cobalt (Co), chromium (Cr), copper (Cu), iron (Fe), lanthanum (La), lithium (Li), manganese (Mn), molybdenum (Mo), nickel (Ni), lead (Pb), rubidium (Rb), tin (Sn), strontium (Sr), titanium (Ti), vanadium (V), and zinc (Zn). A solution containing four internal standard (IS) elements (germanium (Ge), scandium (Sc), rhodium (Rh), and rhenium (Re)) (SCP SCIENCE, Clark Graha, Baie D’Urfé, Canada) was continuously delivered and mixed online with the liquid sample stream. Internal standards were used to compensate for variations in sample introduction efficiencies, instrument drift, and sample matrix effects. Internal standards were added to all solutions at the same concentration level, including blank calibration standards and samples. The final concentration was 40 µg L^−1^. The calibration curves ranged from 0.05 to 1000 µg L^−1^.

The samples were diluted with ultrapure water (Millipore, El Paso, TX, USA) at two different dilution factors (1:2 and 1:4). A univariate optimization of the dilution factor was carried out (Section 3.1.1). According to the internal standardization procedure, the ratio of the signal for the analytes to that for the internal standard was plotted against the analyte concentration divided by the concentration of the internal standard.

### 2.4. Calculation of the Limit of Detection (LOD) and Quantification (LOQ)

The limits of detection (*LOD*) and quantification (*LOQ*) were calculated according to the 3*s_b_* and 10*s_b_* criteria, respectively.

(1) $$LOD=\frac{3s_{b}}{m}$$

(2) $$LOQ=\frac{10s_{b}}{m}$$

where *s_b_* is the standard deviation of 10 consecutive blank measurements and *m* is the slope of the calibration line.

### 2.5. Matrix Effects Study

In order to evaluate the extent of the matrix interferences, a recovery factor was calculated according to Equation (3):

(3) $$Recovery=\frac{{Intensity}_{spikedsample}-{Intensity}_{Non-spikedsample}}{{Intensity}_{50ppbaqueousstandard}}\times 100$$

To determine the analyte recoveries, a sample aliquot was spiked at a known analyte concentration (i.e., 100 µg L^−1^) with the multielemental standard. Non-spiked samples were taken as blanks. 

### 2.6. Method Validation

Once the best dilution factor and experimental conditions were selected, a complete in-house validation according to the Eurachem guidelines [28] was carried out. 

The linearity of the method was evaluated by performing calibration using eight elemental concentrations ranging from 0.05 to 1000 µg L^−1^. Five replicates were measured for each calibration solution. Coefficients of determination (R^2^) higher than 0.995 were obtained for the different calibration curves.

Trueness was assessed by a recovery study. A PF sample was spiked with a 50 µg L^−1^ multielemental solution, and five sub-samples were measured on four different days. The recovery rate was calculated as the ratio between the found and the spiked concentration values (Equation (3)).

The contribution of trueness to the uncertainty (*u_t_*) was calculated based on Equation (4):

(4) $$u_{t}=\frac{100\times u_{Rec}}{C_{Theoretical}}$$

where *C_Theoretical_* is the theoretical added concentration. The uncertainty of the measurement results on the spiked sample (*u_Rec_*) combined the difference between the experimental value and the theoretical added concentration values (*RMS_Bias_*) with the combined uncertainty of the spiked sample solution (*u_Conc_*) and the uncertainty of the sample preparation (*u_Prep_*):

(5) $$u_{Rec}=\sqrt{{RMS}_{bias}^{2}+u_{CRecovery}^{2}}$$

(6) $$u_{CRecovery}=\sqrt{u_{Conc}^{2}+u_{Prep}^{2}}$$

Five sub-samples measured on four different days, using five different calibration curves, were used to determine the uncertainty contributions related to the repeatability and intermediate precision. One-way ANOVA was used to estimate the repeatability and intermediate precision as within-group and between-group standard deviations, respectively. The relative standard uncertainty contributions related to the repeatability and intermediate precision were obtained by applying Equations (7) and (8), repectively:

(7) $$u_{rep}=\frac{100}{C_{Theoretical}}\sqrt{\frac{{RSD}_{rep}^{2}}{n_{rep}}}$$

(8) $$u_{ip}=\frac{100}{C_{Theoretical}}\sqrt{\frac{{RSD}_{ip}^{2}}{n_{days}}}$$

where *C_Theoretical_* is the theoretical added concentration, *RSD_rep_* is the repeatability, *n_rep_* is the number of replicates, *RSD_ip_* is the intermediate precision, and *n_days_* is the number of days. Moreover, the relative standard uncertainty related to the trueness contribution was estimated by applying Equation (8).

The contributions of the repeatability, intermediate precision, and trueness were considered for the calculation of the expanded uncertainty (*U*) of the measurements.

(9) $$U=k\cdot \sqrt{u_{rep}^{2}+{u_{ip}^{2}+u_{t}^{2}}_{}^{}}$$

where *U* is the expanded relative uncertainty, *k* is the coverage factor (*k* = 2), *u_rep_* is the relative standard uncertainty of repeatability, *u_ip_* is the relative standard uncertainty of intermediate precision, and *u_t_* is the relative standard uncertainty of trueness.

## 3. Results and Discussion

### 3.1. Method Optimization

One of the main limitations for the multielemental quantification of PF samples was the limited sample volume available for their analysis. Typically, liquid flow rates used with conventional sample introduction systems could reach values close to 1 mL min^−1^. In the present work, it was necessary to lower the sample flow rates to 100 µL min^−1^. Furthermore, due to the complexity of the PF matrix, the developed analytical method was optimized to mitigate matrix effects and spectral interferences.

#### 3.1.1. Matrix Effects

Peritoneal fluids are characterized by a rather complex composition, consisting of various organic and inorganic components, such as proteins and dissolved salts (e.g., Na, K, Ca, Mg, Cl, P, or S) [27]. The sample matrix could modify the aerosol characteristics, the amount of analyte delivered to the plasma, and the thermal plasma characteristics [29]. Consequently, the matrix could cause a modification of the signal with respect to a standard, degrading the accuracy of the results. To evaluate the impact of the sample dilution on the extent of matrix interferences and sensitivity, two dilution factors (1:2 and 1:4) were tested on five randomly selected peritoneal fluids spiked with a known concentration (100 µg L^−1^) of the multielemental standard (SCP33MS).

**Figure 1:**
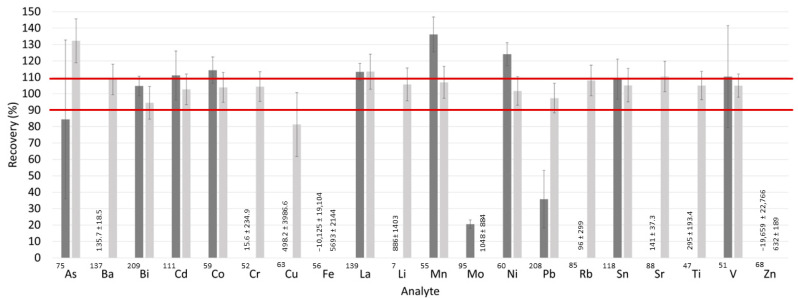
Recovery values for five spiked peritoneal fluid samples (at 100 µg L^−1^), calculated from 5 replicate measurements. Five randomly selected PF samples were selected to obtain recoveries. The recovery values were obtained for samples diluted with ultrapure water at ratios of 1:2 (dark grey bars) and 1:4 (light grey bars). The measurements were performed by ICP-MS/MS. The experimental conditions are available in Table S1 in the Supplementary Materials. No collision gas was added. The mean (bars) and standard error of the mean (error bars) are shown. For those elements with average recovery values outside the range displayed on the axis, numerical values are provided.

The recoveries calculated using Equation (3) deviated from 100% for many analytes (^75^As, ^137^Ba, ^52^Cr, ^63^Cu, ^56^Fe, ^7^Li, ^55^Mn, ^95^Mo, ^60^Ni, ^208^Pb, ^85^Rb, ^88^Sr, ^47^Ti, and ^68^Zn) when a 1:2 dilution factor was applied, as shown in Figure 1. The recoveries for ^75^As, ^52^Cr, ^56^Fe, ^95^Mo, ^208^Pb, and ^68^Zn were below 100%, while for ^63^Cu, ^137^Ba, ^7^Li, ^55^Mn, ^60^Ni, ^88^Sr, and ^47^Ti the recoveries were above 100%. The impact of matrix effects was reduced when a higher dilution factor (1:4) was used (Figure 1). A tolerance interval of ±10% was established, with good recoveries lying within the range of 90–110%. The recoveries for ^137^Ba, ^111^Cd, ^59^Co, ^52^Cr, ^7^Li, ^55^Mn, ^60^Ni, ^208^Pb, ^85^Rb, ^118^Sn, ^88^Sr, ^47^Ti, and ^51^V were within this interval. However, the recoveries obtained for ^75^As, ^63^Cu, ^56^Fe, ^95^Mo, and ^68^Zn were outside of the tolerance interval. The recovery for ^63^Cu was below 100%, while for ^75^As, ^56^Fe, ^95^Mo, and ^68^Zn it was above 100%. Therefore, the 1:4 dilution factor was selected. 

#### 3.1.2. Spectral Interferences

Elements with unacceptable recovery values are shown in Figure 1. Some of these elements may experience spectral interferences caused by ions with the same mass as the analyte ions (Table 1). These spectral interferences could be induced by polyatomic ions originating from the plasma gas or matrix components. For instance, the presence of chloride in the samples could lead to the spectral overlap of ^35^Cl^40^Ar on the ^75^As isotope [30]. To mitigate spectral interferences, a collision cell positioned before the analyzer quadrupole could be employed. The basic concept involves using a non-reactive gas, such as He, to stimulate ion–molecule collisions, as polyatomic ions have a larger ionic cross-section area than analyte ions with the same mass-to-charge ratio, thereby promoting collisions between He atoms and polyatomic ions.

**Figure 2:**
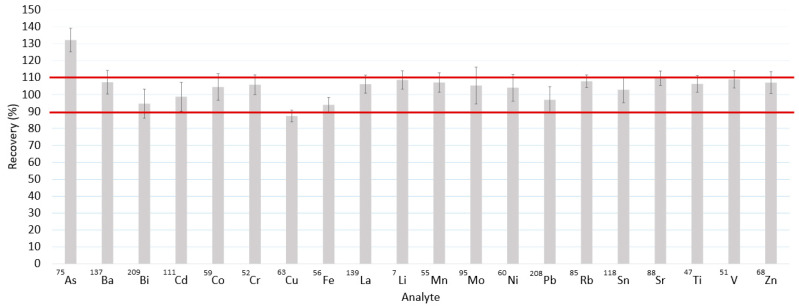
Recovery values for five spiked peritoneal fluid samples (at 100 µg L^−1^), calculated from 5 replicate measurements. Five randomly selected PF samples were selected to obtain recoveries. The recovery values were obtained for samples diluted 1:4 with ultrapure water. The measurements were performed by ICP-MS/MS. The experimental conditions are available in Table S1 in the Supplementary Materials. He was used as a collision gas. The mean (bars) and standard error of the mean (error bars) are shown.

The addition of a collision gas minimized spectral interferences for ^63^Cu, ^56^Fe, and ^68^Zn (Figure 2), leading to improved recoveries compared to those obtained without He (Figure 1). For example, for the ^56^Fe isotope, which is spectrally interfered with by ^40^Ca^16^O and ^40^Ar^16^O (Table 1), the recovery factor decreased from values near 6000% to values equal to 95%. However, the addition of the collision gas did not improve the recovery value for arsenic, which may be attributed to other processes, such as plasma charge transfer reactions between ^75^As and the carbon present in the sample [31].

### 3.2. Analytical Figures of Merit

The limits of detection (LOD) and quantification (LOQ) were calculated for each analyte on four different days by applying Equations (1) and (2). Table 2 shows the LOD and LOQ values in He and no-gas modes. Generally, the LOD and LOQ values were lower in no-gas mode than in He mode for most elements. However, it is important to note that the addition of a collision gas has a dilution effect and, therefore, degrades the LOD and LOQ. Moreover, for elements with spectral interferences, the LOD and LOQ values in no-gas mode were higher than those in He mode, due to an intensification in the standard deviation of the blank signal caused by spectral interferences in no-gas mode. 

**Figure 3:**
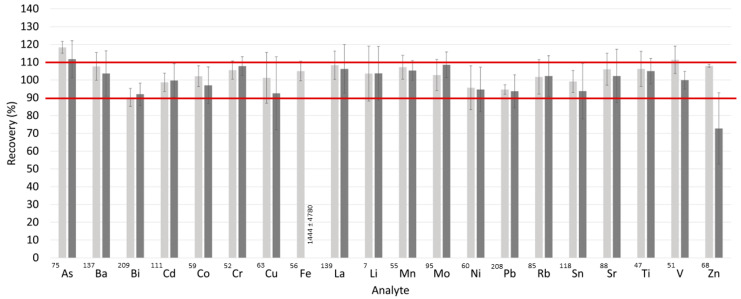
Recovery values for five spiked peritoneal fluid samples (at 100 µg L^−1^), calculated from 5 replicate measurements. Five randomly selected PF samples were selected to obtain recoveries. The recovery values were obtained for samples diluted 1:4 with ultrapure water. The measurements were performed by ICP-MS/MS. The experimental conditions are available in Table S1 in the Supplementary Materials. He was used as a collision gas (light grey), or no-gas mode was employed (dark grey). The mean (bars) and standard error of the mean (error bars) are shown. For those elements with average recovery values outside the range displayed on the axis, numerical values are provided.

An interday validation test was carried out to evaluate the accuracy by means of a recovery study (Equation (3)). A randomly selected sample of PF was spiked and analyzed. Recovery was determined on four different days in He and no-gas modes. The mean values of the recoveries and the standard error of the mean (SEM) are shown in Figure 3. For the no-gas mode, the mean values of the recoveries were around 100% for all of the isotopes, except for those presenting spectral interferences (^52^Cr, ^56^Fe, ^63^Cu, ^68^Zn, and ^75^As). In contrast, in He mode, all of the recoveries—except that for ^75^As—lay within the tolerance interval (i.e., 100 ± 10%). Additionally, the accuracy of the analyte concentration determination was evaluated by comparing the results obtained using no-gas mode with those obtained using a collision gas (He). Table 3 shows the results for those elements above the limit of detection in at least one measurement method. Moreover, the concentrations for the elements subject to spectral interference were not compared. Although there were few instances where the concentrations for both methods were above the LOQ, both measurement methods provided similar concentration values in most cases (Table 3).

### 3.3. Complete Method Validation

According to the results previously shown, the developed method allows the accurate analysis of PF, with the use of a 1:4 sample dilution factor and the optional addition of a collision gas to minimize both non-spectral and spectral interferences. An in-house validation and uncertainty estimation was carried out. For those elements affected by non-spectral interference, the no-gas mode was used to achieve the lowest detection and quantification limits. However, for ^52^Cr, ^56^Fe, ^63^Cu, and ^68^Zn, helium was used as a collision gas. The expanded uncertainty values for most of the elements in both no-gas and He modes were approximately 15% (Table 4 and Table 5), with the trueness assessment representing 75–85% of the uncertainty. It should be noted that a recovery study was conducted, as no certified reference material was available; thus, the trueness uncertainty included the contribution of bias, as well as the uncertainty of the multielemental stock solution and the sample preparation process.

### 3.4. Sample Analysis under Optimal Conditions

The elemental contents of 20 PF samples were determined by ICP-MS/MS. To perform the analysis, a 1:4 sample dilution factor was applied. It is important to note that He mode (collision gas) was applied for those elements suffering from spectral interferences: ^52^Cr, ^63^Cu, ^56^Fe, and ^68^Zn. Unfortunately, accurate measurements of arsenic were not possible under the experimental conditions used.

In no-gas mode (Table 6), the analytes that were detected in 100% of the samples were ^60^Ni, ^85^Rb, ^88^Sr, and ^47^Ti. Lithium, in turn, was present in 45% of the samples at levels above the LOD. Some PTMEs—such as ^95^Mo, ^209^Bi, ^111^Cd, and ^59^Co—were found in a high percentage of samples (40–65%), while others—such as ^55^Mn, ^208^Pb, and ^51^V—were found in a very high percentage of samples (70–80%). Table 7 shows that the four elements measured in He mode were present in 100% of the samples.

In terms of concentrations, the minor analytes were ^59^Co and ^139^La, which were present at concentrations below 1 µg L^−1^; ^209^Bi, ^111^Cd, ^52^Cr, ^55^Mn, ^95^Mo, ^60^Ni, ^208^Pb, ^118^Sn, and ^51^V were present at concentrations ranging from 1 to 10 µg L^−1^; ^137^Ba, ^7^Li, ^85^Rb, ^88^Sr, and ^47^Ti presented concentration levels up to 151 µg L^−1^. The major analytes found in PF samples were ^63^Cu, ^56^Fe, and ^68^Zn.

### 3.5. Essential and Non-Essential Elements 

In this section, elements are grouped as essential and non-essential to the organism and according to the concentrations found in PF samples. The roles of the essential elements in the organism, along with toxicological aspects such as the recommended daily allowance (RDA) and the tolerable upper intake level (UL), are detailed. For non-essential elements, safe daily levels and dietary consumption limits are summarized. Additionally, the elemental compositions found in the 20 PF samples are compared with the data obtained for biological fluids from previously published works, with a focus on their relevance to gynecological and reproductive health.

#### 3.5.1. Elements Found at Major Concentrations in PF Samples

According to the concentrations found in PF samples, the essential major analytes were Zn, Fe, and Cu, as shown in Table 7. Zinc is an essential element required for the proper functioning of enzymes involved in protein maintenance and the regulation of gene expression. The recommended daily dose for Zn is 8 mg, while the UL is 40 mg [32]. High serum Zn levels have been linked to conditions such as polycystic ovary syndrome (PCOS), dysmenorrhea, and endometriosis. Serum levels of Zn were significantly higher in PCOS patients compared to controls (0.92 versus 0.77 µg mL^−1^, respectively) [33]. In the analyzed PF samples, Zn was found in 100% of the samples, with an average value of 6889 ± 13,385 µg L^−1^ (Table 7). In this context, the Zn concentration in the analyzed PF samples was higher than in serum [33]. 

Iron is another crucial element found in metalloproteins such as myoglobin and hemoglobin. The RDA of Fe for an adult woman is 18 mg per day, while the UL is 45 mg [32]. Higher iron concentration, transferrin saturation, and ferritin concentration were found in the PF of endometriosis patients than in the control group (59 and 49 mg mL^−1^, respectively) [34]. Fe was found in the follicular fluid (FF) in women with diminished ovarian reserves (DOR, 1.56 mg L^−1^) and in healthy controls (1.50 mg L^−1^) [35]. In this work, we found that Fe was present in 100% of the PF samples, with a mean value of 3118 ± 5123 µg L^−1^ (Table 7), which was significantly below those reported in the literature [34]. Conversely, the content of Fe in PF was higher as compared to FF [35]. 

Copper is a component of many metalloenzymes with oxidase activity involved in the reduction of molecular oxygen. The RDA for Cu is 900 µg, with UL levels of 10,000 µg [32]. Moreover, Cu has been considered as a potential target for cancer treatment because of its excessive elevated levels in malignant tissues, together with properties that promote angiogenesis, cancer growth, and metastasis. Aberrant elevated serum Cu concentrations have been found in malignant breast cancer tumors compared to controls (1252.20 ± 150.90 versus 964.95 ± 70.30 µg L^−1^, respectively) [36]. Cu was detected in 100% of the analyzed PF samples, with concentration levels (Table 7) similar to those found in serum [36].

#### 3.5.2. Elements Found at Intermediate Concentrations in PF Samples

The essential elements that were present at intermediate concentrations in PF (i.e., levels up to 151 µg L^−1^) were Rb and Li (Table 6). Previous studies have shown that Rb is present in the whole-blood samples of pregnant women (control group: 1867.40 ± 392.50 µg L^−1^) and women who have suffered a miscarriage (1787.69 ± 384.92 µg L^−1^), with no significant differences. Interestingly, Rb was negatively associated with spontaneous abortion [37]. Rb was present in all 20 PF samples included in the present study, with an average concentration of 150.7 ± 57.9 µg L^−1^ (Table 6)—significantly lower than the values found in whole blood, as previously described [37]. Li, in turn, may have essential functions in animals, but it is not known whether there is a human requirement [1]. Toxicity from dietary Li is unknown, with an intake from dietary sources of approximately 100 µg per day. Li was found in FF in PCOS and in control women at similar concentration levels (2.75 ± 0.52 and 2.78 ± 0.67 μg L^−1^, respectively) [38]. In our study, Li was detected in 45% of the samples, with a higher concentration (12.7 ± 9.6 μg L^−1^, Table 6) than reported in FF [38]. 

#### 3.5.3. Elements Found at Minor Concentrations in PF Samples

As regards minor essential elements in PF, Mo, Cr, Mn, Ni, and V were found at concentrations between 1 and 10 µg L^−1^ (Table 6 and Table 7), while Co was present at concentrations below 1 µg L^−1^ (Table 6). Mo is a cofactor of enzymes involved in the catabolism of sulfur amino acids, purines, and pyrimidines. The RDA of Mo ascends to 45 µg, while the UL is 2000 µg [32]. Mo levels have been reported in various biological samples, including urine, FF, serum, and placenta. Regarding the placenta, toxic and essential elements in this biological matrix, together with its relation to birth outcomes in women exposed to a low-toxic-metal environment, were investigated. The median Mo concentration value found in the placenta was 5.3 µg L^−1^ [39], while the concentration levels in the sera and FF of women attending an in vitro fertilization (IVF) center were 2.94 ± 7.26 and 1 ± 1.17 µg L^−1^, respectively [40]. In contrast to the previous data, Mo was detected in 40% of the PF samples, with only one sample above the LOQ, at a concentration of 17.9 µg L^−1^ (Table 6).

Cr is an essential nutrient that promotes the action of insulin, thereby influencing the carbohydrate, lipid, and protein metabolism [1]. Recent reports indicate that the RDA of Cr for adults is 20–35 μg per day [32]. Studies on environmental exposure revealed that placental Cr contents did not differ significantly between environmentally exposed women and the control group (244.19 versus 229.08 ng per wet weight, respectively) [41]. Regarding IVF, Cr in FF was positively correlated with the number of oocytes in the MII stage, with a mean concentration of 2.46 ± 2.66 µg L^−1^ [40]. In the present study, Cr was detected above the LOD in 100% of the PF samples, with a mean concentration of 6.6 ± 7.9 µg L^−1^ (Table 7). Cr appeared to be present at higher concentrations in PF compared to the placenta and FF samples [40,41].

Manganese is a trace element involved in bone formation and is present in enzymes involved in the metabolism of amino acids, cholesterol, and carbohydrates, with a daily recommended dose of 1.8 mg and a UL of 11 mg [32]. Increased levels of Mn in maternal whole blood (MWB) or umbilical cord blood (UCB) could be associated with environmental exposure. The MWB level of Mn was 54.98 ± 22.98 µg L^−1^, while the UCB Mn concentration was 78.75 ± 30.53 µg L^−1^, with a quadratic curvilinear relationship reported between Mn and birth size [42]. The Mn concentrations in the analyzed PF samples (Table 6) were lower than the concentration levels found in whole blood [42], with a mean concentration of 4.6 ± 4 µg L^−1^.

No biological function in humans has been found for Ni and V, although Ni has been found to potentially serve as a cofactor of metalloenzymes. The ULs for Ni and V are 1 and 1.8 mg per day, respectively. A study on women undergoing IVF found that the Ni levels in serum and follicular fluid (FF) were 1.61 ± 3.96 and 2.76 ± 2.43 µg L^−1^, respectively. In our study, 100% of the PF samples contained Ni, and the mean concentration was 5.4 ± 3.2 µg L^−1^ (Table 6). The V levels found in serum and FF in the previously mentioned study were 0.53 ± 0.35 µg L^−1^ and 0.49 ± 0.26 µg L^−1^, respectively [40]. Similarly, V levels were found to be higher in PF compared to serum and FF, with a detection rate of 70% and a mean concentration of 7.4 ± 1.9 µg L^−1^ (Table 6). 

Cobalt, on the other hand, is a critical component of hydroxycobalamin (vitamin B12), which is essential for red blood cell production. In this case, the RDA for B12 is 2.4 μg per day [32]. Although the mean values for cobalt in biological fluids are typically low (0.16 µg L^−1^ for serum and 0.40 µg L^−1^ for urine) [43], our study found that 40% of the PF samples contained cobalt, with a mean value of 2.3 ± 0.3 µg L^−1^ (Table 6). It should be noted that Fe and Co in excess may promote oxidative stress and cause tumor formation [44].

#### 3.5.4. Non-Essential Hhuman Elements

Various elements, including Ba, Bi, Cd, La, Li, Pb, Sn, Sr, and Ti, are considered non-essential for human beings; hence, there is no recommended dietary allowance (RDA) for these elements. Instead, safe daily levels and limits for dietary consumption are provided based on safety considerations. Despite their non-essential nature, some of these elements—such as Ba, Bi, La, Li, Sr, and Sn—have been linked to gynecological health, but the available data on their content in biological fluids and their relationships with gynecological health are limited. According to the concentrations found in PF samples, the non-essential analytes found at concentrations up to 64 µg L^−1^ were Ti, Ba, and Sr (Table 6).

Titanium exposure has been a concern due to its association with several adverse health effects, but the understanding of the effects of Ti on gynecological and reproductive health remains limited. An adult weighing 50 kg may consume approximately 10–35 mg of TiO_2_ through their diet [45]. Blood Ti levels were investigated in women who delivered normal-birth-weight infants (control group) and women who delivered low-birth-weight infants (case group). The median total blood concentration of Ti in the case group was significantly higher than in the control group (134 vs. 129 μg L^−1^) [46]. In FF, Ti was found to be significantly higher in healthy controls (179.06 μg L^−1^) compared to women with DOR (149.78 μg L^−1^) [35]. For PF samples, we found that Ti was present in 100% of the samples, with a mean concentration of 64.3 ± 68.9 μg L^−1^ (Table 6), which is different from the concentrations found in total blood and FF [35,46].

For Sr, there is a general lack of information available, but it was found in the FF of women with DOR (26.02 μg L^−1^) and healthy controls (36.48 μg L^−1^) [35]. In PF, we found that Sr was detected in all PF samples, with a mean value of 33.5 ± 11.1 μg L^−1^ (Table 6), which is similar to the concentrations found in FF samples. The Ba intake from food, water, and air is estimated to range from about 0.7 to 1.9 mg per day, with food being the primary source of intake for those who are not occupationally exposed [47]. In women undergoing IVF, Ba was found in the serum (17.11 ± 43.4 µg L^−1^) and FF (10.34 ± 18.57 µg L^−1^) [40]. Our results demonstrated the presence of Ba in 85% of the PF samples, with a mean value of 21.7 ± 31.8 µg L^−1^ (Table 6), similar to the values previously described as being found in serum [40].

Regarding minor non-essential elements, Pb, Bi, Sn, and Cd were found in PF samples at levels in between 1 and 10 µg L^−1^ (Table 6). The Food and Drug Administration (FDA) limits dietary Pb consumption to 8.8 μg per day for females of childbearing age [48]. There is no safe blood concentration of Pb, as even levels as low as 5 μg dL^−1^ could be associated with health problems [49]. Furthermore, it is known that as lead exposure increases, the range and severity of symptoms and effects grow. For example, occupationally exposed women had significantly higher levels of Pb in their blood (2.73 ± 2.39 µg dL^−1^) compared to those who were not occupationally exposed (1.25 ± 2.10 µg dL^−1^) [50]. Smoking increases Pb levels in the endometrium [14]. In IVF patients, Pb levels were 3.01 ± 2.77 µg L^−1^ in serum and 7.42 ± 32.51 µg L^−1^ in FF [40]. Compared to the previous data, in PF samples, Pb was detected in 80% of the samples, with a mean value of 6.3 ± 5.9 µg L^−1^ (Table 6)—similar values to those found in FF samples [40].

Bismuth was detected in 65% of the PF samples (Table 6). However, the long-term use of Bi may result in side effects and toxicity to human beings, depending on the nature and amount of the absorbed Bi species. It is not clear why selected individuals develop Bi toxicity. As for Bi in biological fluids, we did not find analytical data regarding Bi and gynecological health [51]. In the case of Sn, the main adverse effect on humans caused by excessive levels of tin in canned beverages (above 150 mg kg^−1^) or other canned foods (above 250 mg kg^−1^) is acute gastric irritation. Approximately 5% of Sn is absorbed from the gastrointestinal tract, distributed in the body, and then excreted by the kidneys. An excess of 130 mg per day could accumulate in the liver and kidneys, causing skin and eye irritation, cholangitis of the lower biliary tract, hepatotoxicity, and neurotoxicity [52]. Sn was found above the LOD in a 75% of the PF samples, with a mean concentration of 3.8 ± 2.1 μg L^−1^ (Table 6).

Cadmium is a PTME that has been widely studied due to its estrogenic activity, which has been linked to the development of estrogen-dependent diseases such as endometrial and breast cancers, endometriosis, and spontaneous abortions [6,11]. Safe daily levels of Cd intake should be kept below 30 µg per person [53]. Endometriosis was demonstrated to be associated with Cd exposure. The blood levels of Cd in women with endometriosis were significantly higher than those in a group of healthy individuals (0.53 vs. 0.46 µg L^−1^, respectively) [54]. Conversely, low levels of Cd in blood from women with uterine myomas (0.33–3.5 µg L^−1^) were correlated with its contents in the uterus, and with significantly decreased estradiol (E2) concentrations in sera [55]. Within this study, Cd was present in 45% of the PF samples (Table 6). The mean value found within the framework of the present study (2.8 ± 1.5 μg L^−1^) was similar to the Cd levels found in blood of women with uterine myomas [55].

Finally, a lack of general information about La was evidenced, and no available data were found concerning La in biological fluids. However, 80% of the PF samples contained La, with a mean concentration of 0.6 ± 1.1 μg L^−1^ (Table 6).

Overall, this study identified the elemental contents of 19 analytes in PF, including essential and non-essential elements such as trace and ultra-trace PTMEs. While some analytes were found at similar concentrations to those reported in whole blood, serum, follicular fluid, and urine (Rb, Cd, Pb, Ti, and Ba), others were found at different amounts (Fe, Cr, Zn, Mn, Mo, V, Ni, and Li). This suggests that PF is a unique biological fluid that cannot be directly compared to other matrices. Nonetheless, the developed method of analysis provides a new approach to diagnose and predict the health risks of women with gynecological pathologies, and to assess women’s health—including reproductive health.

## 4. Conclusions

This is the first work where an analytical method for the determination of 19 analytes by ICP-MS/MS in PF samples has been developed and validated. The methodology was found to be highly suitable for performing accurate determination of elements of interest such as ^137^Ba, ^209^Bi, ^111^Cd, ^59^Co, ^52^Cr, ^63^Cu, ^56^Fe, ^139^La, ^7^Li, ^55^Mn, ^95^Mo, ^60^Ni, ^208^Pb, ^85^Rb, ^118^Sn, ^88^Sr, ^47^Ti, ^51^V, and ^68^Zn. The main advantages of the developed procedure are as follows: (i) the amount of sample required to perform the analysis was reduced as it was aspirated at a low liquid flow; (ii) the application of an appropriate dilution factor allows the mitigation of matrix effects without degradation of the limits of detection and quantification; (iii) accurate determination of ^52^Cr, ^63^Cu, ^56^Fe, and ^68^Zn was possible through the addition of a collision gas; (iv) the method’s expanded uncertainty was lower than 15% for the 19 analytes; and (v) it avoids the use of toxic reagents, thereby lowering the cost and time of analysis and aligning the method with the principles of green chemistry.

This advanced research method could help individuals to identify and understand their health risks and monitor their health status over time. Specifically, this novel method could be employed as an advance routine analysis method for the analysis of PF, opening the possibility of using metal concentrations as an indicator of women’s fertility status, as well as to diagnose and prognose related gynecological pathologies. However, the main limitation of this study is the analysis of a limited number of PF samples (20 PF samples) that may not be sufficient to obtain conclusive results. The present work could be considered as a preliminary examination, and ongoing research with a wider study group is currently being carried out in our laboratories. The results of this extended study will be the subject of a forthcoming publication, which will provide more conclusive evidence regarding the efficacy of essential and non-essential elements such as trace and ultra-trace PTMEs for assessing women’s reproductive health.

## Figures and Tables

**Table 1:** ICP-MS spectroscopic interferences found when analyzing peritoneal fluids for isotopes of various elements.

Isotope	Abundance (%)	Interference from the Matrix/Argon Plasma
^52^Cr	83.8	^40^Ar^12^C^+^, ^35^Cl^17^O^+^
^56^Fe	91.7	^40^Ca^16^O^+^,^40^Ar^16^O^+^
^63^Cu	69.2	^36^Ar^12^C^14^N^1^H^+^, ^14^N^12^C^37^Cl^+^, ^16^O^12^C^35^Cl^+^
^68^Zn	18.6	^40^Ar^14^N_2_^+, 35^Cl^16^O^17^O^+, 64^S_2_^+, 38^Ar^32^S^+^
^75^As	100	^23^Na^12^C^40^Ar, ^12^C^31^P^16^O_2_^+^, ^40^Ar^35^Cl^+^, ^36^Ar^39^K^+^

**Table 2:** Limits of detection (LOD) and quantification (LOQ) in He and no-gas modes (µg L^−1^), obtained on four different days. The measurements were performed by ICP-MS/MS. The experimental conditions are available in Table S1 in the Supplementary Materials. He was used as a collision gas.

	LOD (µg L^−1^)		LOQ (µg L^−1^)	
	No Gas	He Gas	No Gas	He Gas
^75^As	1.7	1.9	6	6
^137^Ba	0.7	1.1	2	4
^209^Bi	0.16	0.2	0.5	0.8
^111^Cd	0.2	0.45	0.7	1.5
^59^Co	0.19	0.2	0.6	0.9
^52^Cr	0.3	0.5	1.1	1.6
^63^Cu	3	2	9	7
^56^Fe	60	4	200	12
^139^La	0.05	0.14	0.18	0.5
^7^Li	0.3	1.6	1.0	5
^55^Mn	0.3	0.7	1.0	2
^95^Mo	1.8	0.4	6	1.3
^60^Ni	0.6	1.0	1.9	3
^208^Pb	0.5	0.2	1.7	0.8
^85^Rb	0.14	0.5	0.4	1.7
^118^Sn	0.4	0.4	1.5	1.3
^88^Sr	1.1	1.1	4	4
^47^Ti	2	6	7	18
^51^V	0.7	0.3	2	1.0
^68^Zn	14	6	47	19

**Table 3:** Analyte concentrations in five peritoneal fluid samples measured in He and no-gas modes. Concentrations are expressed in μg L^−1^. The measurements were performed by ICP-MS/MS. The experimental conditions are available in Table S1 in the Supplementary Materials. He was used as a collision gas.

Analyte	Peritoneal Fluid Sample				
	Sample 1	Sample 2	Sample 3	Sample 4	Sample 5
^137^Ba (No Gas)	2.8 ± 0.3	2.7 ± 0.4	18.4 ±1.0	11.0 ±1.4	19.7 ± 0.8
^137^Ba (He)	0.58 ± 0.15	<LOQ (4)	16.9 ± 1.4	13.8 ± 0.8	19.2 ± 0.7
^209^Bi (No Gas)	0.34 ± 0.07	0.34 ± 0.12	0.77 ± 0.13	0.20 ± 0.02	0.17 ± 0.03
^209^Bi (He)	<LOQ (0.8)	<LOQ (0.8)	0.85 ± 0.09	0.49 ± 0.03	<LOQ (0.8)
^111^Cd (No Gas)	<LOQ (0.5)	<LOQ (0.5)	<LOQ (0.5)	<LOQ (0.5)	4.4 ± 0.4
^111^Cd (He)	<LOQ (1.5)	<LOQ (1.5)	<LOQ (1.5)	<LOQ (1.5)	5.3 ± 1.7
^59^Co (No Gas)	<LOQ (0.3)	<LOQ (0.3)	<LOQ (0.3)	<LOQ (0.3)	2.50 ± 0.06
^59^Co (He)	<LOQ (0.9)	<LOQ (0.9)	<LOQ (0.9)	<LOQ (0.9)	1.9 ± 0.5
^139^La (No Gas)	<LOQ (0.14)	<LOQ (0.14)	0.21 ± 0.03	0.24 ± 0.05	3.22 ± 0.16
^139^La (He)	<LOQ (0.5)	<LOQ (0.5)	<LOQ (0.5)	<LOQ (0.5)	2.80 ± 0.09
^7^Li (No Gas)	2.70 ± 0.07	<LOQ (0.9)	10.8 ± 0.2	<LOQ (0.9)	<LOQ (0.9)
^7^Li (He)	<LOQ (5)	<LOQ (5)	9.8 ± 0.5	<LOQ (5)	<LOQ (5)
^55^Mn (No Gas)	1.06 ± 0.04	2.18 ± 0.08	<LOQ (0.7)	<LOQ (0.7)	3.70 ± 0.11
^55^Mn (He)	<LOQ (2)	<LOQ (2)	<LOQ (2)	<LOQ (2)	<LOQ (2)
^60^Ni (No Gas)	3.0 ± 0.3	4.4 ± 0.6	5.10 ± 0.12	11.1 ± 0.4	6.8 ± 0.4
^60^Ni (He)	<LOQ (3)	<LOQ (3)	5.5 ± 1.1	4.3 ± 0.9	6.2 ± 0.8
^208^Pb (No Gas)	<LOQ (1.7)	<LOQ (1.7)	4.84 ± 0.08	2.77 ± 0.04	<LOQ (1.7)
^208^Pb (He)	0.98 ± 0.07	1.57 ± 0.11	4.64 ± 0.18	2.49 ± 0.16	1.33 ± 0.12

**Table 4:** Relative standard uncertainty contributions and expanded relative uncertainty for measurements of metal contents in peritoneal fluid samples in no-gas mode. A PF sample was spiked with a 50 µg L^−1^ multielemental solution, and five sub-samples were measured on four different days. The recovery values were obtained for samples diluted 1:4 with ultrapure water. The measurements were performed by ICP-MS/MS. The experimental conditions are available in Table S1 in the Supplementary Materials. No-gas mode was used.

	^137^Ba	^209^Bi	^111^Cd	^59^Co	^139^La	^7^Li	^55^Mn	^59^Mo	^60^Ni	^208^Pb	^85^Rb	^118^Sn	^88^Sr	^47^Ti	^51^V
*u_ip_*	2.7	1.8	3.1	0.8	1.0	1.2	0.8	1.2	0.9	1.4	1.5	1.5	0.9	1.3	1.2
*u_rep_*	1.1	1.1	0.9	1.4	1.4	2.3	1.3	1.7	1.7	1.4	1.7	2.2	1.9	1.8	1.4
*u_t_*	7.5	8.0	7.1	6.7	4.5	8.7	5.1	7.9	6.3	6.3	6.8	8.8	8.1	5.3	7.7
Expanded relative uncertainty (*U*, k = 2)	16.0	16.6	15.6	13.8	9.6	18.2	10.6	16.3	13.1	13.2	14.3	18.4	16.7	11.5	15.8

**Table 5:** Relative standard uncertainty contributions and expanded relative uncertainty for measurements of Cr, Cu, Fe, and Zn contents in peritoneal fluid samples in He mode. A PF sample was spiked with a 50 µg L^−1^ multielemental solution, and five sub-samples were measured on four different days. The recovery values were obtained for samples diluted 1:4 with ultrapure water. The measurements were performed by ICP-MS/MS. The experimental conditions are available in Table S1 in the Supplementary Materials. He was used as a collision gas.

	^52^Cr	^63^Cu	^56^Fe	^68^Zn
*u_ip_*	1.7	1.3	1.3	1.3
*u_rep_*	2.5	1.8	1.6	1.9
*u_t_*	7.3	6.9	8.0	8.2
Expanded relative uncertainty (*U*, k = 2)	15.8	14.5	16.5	17.0

**Table 6:** Trace and ultra-trace metal elements and PTMEs; mean, standard error of the mean (SEM), minimum (min), and maximum (max) values, and percentages of samples above the limit of detection (%), found in 20 peritoneal fluid samples measured in no-gas mode. Samples were diluted 1:4 with ultrapure water. The measurements were performed by ICP-MS/MS. The experimental conditions are available in Table S1 in the Supplementary Materials.

Analyte	Mean ± SEM (μg L^−1^)	Min (μg L^−1^)	Max (μg L^−1^)	% > LOD
^137^Ba	21.7 ± 31.8	2.7	110.7	85
^209^Bi	4.7 ± 7.5	0.6	15.8	65
^111^Cd	2.8 ± 1.5	0.9	4.4	55
^59^Co	2.3 ± 0.3	2.1	2.5	40
^139^La	0.6 ± 1.1	0.2	3.9	80
^7^Li	12.7 ± 9.6	1.6	25.3	45
^55^Mn	4.6 ± 4.0	1.1	14.6	75
^95^Mo	17.9	-	-	40
^60^Ni	5.4 ± 3.2	2.9	14.5	100
^208^Pb	6.3 ± 5.9	1.8	17.9	80
^85^Rb	150.7 ± 57.9	77.2	313.5	100
^118^Sn	3.8 ± 2.1	2.2	7.4	75
^88^Sr	33.5 ± 11.1	9.8	54.1	100
^47^Ti	64.3 ± 68.9	12.2	219	100
^51^V	7.4 ± 1.9	4.3	11.5	70

**Table 7:** Trace and ultra-trace metal elements and PTMEs; mean, standard error of the mean (SEM), minimum (min), and maximum (max) concentration values, and percentages of samples above the limit of detection (%), found in 20 peritoneal fluid samples measured in He mode. Samples were diluted 1:4 with ultrapure water. The measurements were performed by ICP-MS/MS. The experimental conditions are available in Table S1 in the Supplementary Materials.

Analyte	Mean ± SEM (μg L^−1^)	Min (μg L^−1^)	Max (μg L^−1^)	% > LOD
^52^Cr	6.6 ± 7.9	1.7	35.1	100
^63^Cu	1115 ± 1466	304	7221	100
^56^Fe	3118 ± 5123	332	17,832	100
^68^Zn	6889 ± 13,385	169	47,040	100

## Supplementary Materials

The following supporting information can be downloaded at: https://www.mdpi.com/article/10.3390/toxics11050399/s1, Table S1: ICP-MS/MS operating conditions.
