# Supplementary material for: A New Advanced and Validated Method for the Determination of Potentially Toxic Metals and Trace and Ultra-Trace Elements in Peritoneal Fluid to Assess the Health Risks of Women with Gynecological Pathologies
Source: Toxics. 2023 Apr 23;11(5):399. doi: 10.3390/toxics11050399 (PMC10222011; doi:10.3390/toxics11050399)
Supplement: Supplementary file 1 [file toxics-11-00399-s001.zip › toxics-2328999-supplementary.pdf]

Table 1. ICP-MS/MS operating conditions.

Parameter	Values
RF Power (kW)	1.55
Plasma flow (L min^{-1})	15
Auxiliary flow (L min^{-1})	1
Nebulizer flow (L min^{-1})	0.8
Makeup gas (L min^{-1})	0.3
Spray chamber Temperature (°C)	-5
Sampling depth (mm)	8
Liquid flow (µL min^{-1})	100
Scan type	MS/MS
Replicates	6
Collision gas flow rate (mL min^{-1})	3
Sweeps	10
**Measured isotopes**	
^{75}As, ^{137}Ba, ^{209}Bi, ^{111}Cd, ^{59}Co, ^{52}Cr, ^{63}Cu, ^{56}Fe, ^{139}La, ^{7}Li, ^{55}Mn, ^{95}Mo, ^{60}Ni, ^{208}Pb, ^{85}Rb, ^{118}Sn, ^{88}Sr, ^{47}Ti, ^{51}V, ^{68}Zn	
**Internal standard isotopes**	
^{72}Ge, ^{185}Re, ^{103}Rh, ^{45}Sc	
